# Reduced osteoclast-derived apoptotic bodies in bone marrow characterizes the pathological progression of osteoporosis

**DOI:** 10.1038/s41420-023-01434-w

**Published:** 2023-04-26

**Authors:** Yutong Wu, Hongbo Ai, Yuhang Xi, Pengbin Yin, Ying Qu, Jianzhong Xu, Ce Dou, Fei Luo

**Affiliations:** 1 Department of Orthopedics, Southwest Hospital, Third Military Medical University (Army Medical University), Chongqing, 400038 China; 2 Department of Orthopedics, Chinese PLA General Hospital, Beijing, 100853 China; 3 National Clinical Research Center for Orthopedics, Sports Medicine & Rehabilitation, Beijing, 100853 China

**Keywords:** Apoptosis, Mesenchymal stem cells, Osteoporosis, Transport carrier

## Abstract

Osteoporosis is associated with excessive activity of osteoclasts. In bone turn over, most osteoclasts undergo apoptosis after bone resorption and produce a large number of apoptotic bodies (ABs). However, the biological function of osteoclast-derived apoptotic bodies (OC-ABs) in the progression of osteoporosis is still unknow. In our study, we identified a reduction of OC-AB quantity in the bone marrow cavity during the progression of osteoporosis, an apoptotic body-deficient MRL/lpr mice were used to study the pro-osteogenic ability of OC-ABs. Mechanistically, OC-ABs promote osteogenesis of bone mesenchymal stem cells (BMSCs) by activating the downstream mTOR pathway via RANKL-mediated reverse signaling. Moreover, systemic infusion of exogenous OC-ABs effectively delayed the bone loss in ovariectomized (OVX) mice, validated the role of OC-ABs as bone protective factor in the pathogenesis of osteoporosis. Taken together, our study elucidates the biological function of OC-ABs in the pathological progression of osteoporotic bone loss and suggests a potential therapeutic strategy to delay bone loss.

## Introduction

Osteoporosis manifests as a systemic impairment of bone mass, bone strength, and microarchitecture, which increases the risk of fractures [1]. What needs to be vigilant is that the incidence of osteoporosis is as high as 23.1% in women all over the world, mainly due to the rapid decline of postmenopausal estrogen stimulates the excessive activity of osteoclasts [2, 3]. At the cellular level, overactive osteoclasts disrupt the balance of bone resorption and bone formation in bone turn over, causing a progressive net bone loss [4]. Some studies have shown that the insufficient osteogenic ability of bone marrow mesenchymal stem cells (BMSCs) caused by aging acts as an accomplice of bone loss [5]. In addition, targeting the functional activity of osteoclasts is effective and beneficial for the treatment of postmenopausal osteoporosis [6–8].

Osteoclasts are a type of specialized multinucleated macrophages in the bone microenvironment, which are generated from monocytes differentiation stimulated by Receptor Activator for Nuclear Factor-κ B Ligand (RANKL) and Macrophage Colony Stimulating Factor (M-CSF) [9]. Mature osteoclasts secrete factors including cathepsin K, carbonic anhydrase II, and H^+^ to resorb bone tissue, and also play an important role in bone development, growth, repair, and reconstruction [10]. In bone remodeling, the bone resorption activity of osteoclasts and the bone formation of osteoblasts maintain a dynamic balance. However, in osteoporosis, the orchestrated balance is disturbed due to excessive osteoclastic activity, which directly leads to pathological bone loss [11]. Meanwhile, activated osteoclasts mediate a shift in bone homeostasis toward bone resorption, accelerating osteoporosis. Once bone resorption and bone formation are dysfunctional, bone health can be severely compromised, with complications such as osteoporotic facture, osteosclerosis, deformity, or osteodystrophy [12–15].

Osteoclasts have a relative short lifespan of about two weeks, and cycle through the generation and apoptosis in bone remodeling, producing a large number of apoptotic bodies (ABs) [16]. AB is a new class of extracellular vesicles discovered in recent years, with a diameter of 1–5 μm and a monolayer membrane structure [17, 18]. Unlike cellular debris, ABs can remain intact for a period of time while carrying part of their parental nucleic acids, amino acids, and proteins, residing in local or circulating to distant organs [19–21]. What’s more, a growing number of research groups have discovered that ABs can mediate cell-to-cell information transfer and are involved in most biological events, including inflammation, autoimmunity, and cancer, which indicate the value of ABs are worth far more than traditionally considered “garbage bags” [22, 23]. During osteogenesis, calcium, and other minerals are deposited into the bone matrix, which strengthens the material and facilitates its growth. Osteoblasts and osteoclasts collaborate in a symbiotic relationship to orchestrate the remodeling of osseous tissue. We previously showed that OC-ABs regulate bone remodeling via RANKL reverse signaling coupling bone resorption and formation [24–26]. However, the function of OC-ABs under pathological conditions, particularly in osteoporosis, is unknown.

Herein, our study identified a decrease of osteoclast-derived ABs abundance in the bone marrow during osteoporosis progression. Using apoptotic body-deficient MRL/lpr mice and the ovariectomized (OVX) model, we affirmed the pro-osteogenic and bone-protective roles of OC-ABs in osteoporosis. These findings partially explain the difference in efficacy of denosumab and bisphosphonates during osteoporosis treatment, and also provide a reference for the prevention, diagnosis, and treatment of osteoporosis.

## Results

### Bone marrow osteoclast-derived apoptotic bodies quantity decreases in the pathogenesis of osteoporosis in OVX mice

**Fig. 1:**
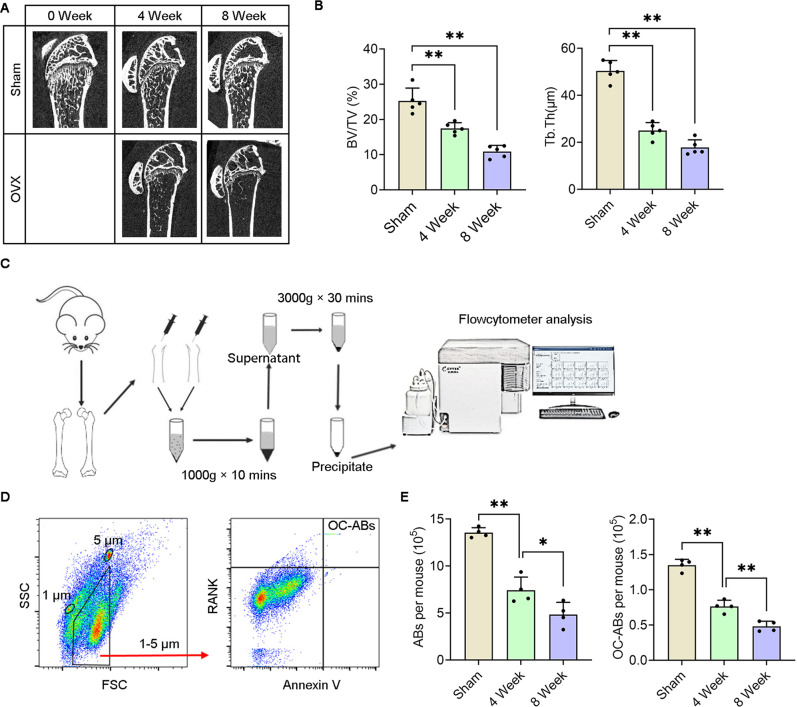
The number of osteoclast-derived apoptotic bodies gradually decreases in the process of osteoporosis. **A** Representative micro-CT images of osteoporotic mice at various stages. **B** Quantification of bone volume fraction and mean thickness of trabecular bone, *n* = 5. **C** Schematic representation of the apoptotic bodies extraction from femoral bone marrow. **D** PE-RANK and FITC-Annexin V label 1–5 μm osteoclast-derived apoptotic bodies. **E** Quantification of total apoptotic bodies and osteoclast-derived apoptotic bodies from femoral bone marrow in each time period, *n* = 4. The data in each panel represent the means ± SD. *p* values were obtained by student’s two-tailed unpaired *t* test, *p < 0.05, **p < 0.01, ns non-significant.

To explore the changes in the bone microenvironment during the pathological progression of osteoporosis, we first established a stable osteoporosis model by ovariectomy. The micro-CT data showed that the ovariectomized mice had decreased bone volume fraction and bone mass over time (Fig. 1A). Software analysis indicated that the estrogen-deficient mice lost about 30 percent of their bone mass at the 2nd week, and even lost half of cancellous bone at the 4th week (Fig. 1B). In addition, with the development of osteoporosis, the thickness of trabecular bone also decreases to half of the normal level which established that the osteoporosis model in this study is stable and reliable (Fig. 1B). Further, we designed the extraction experiment to obtain total osteoclast-derived apoptotic bodies from femurs via a series of density gradient centrifugation (Fig. 1C). In brief, the intact femurs on both sides of the mice were extracted, and the growth plates at both ends were cut along the epiphyseal line. The marrow cavity was repeatedly flushed with 5 mL PBS using a syringe until blanched, and the cell suspension was then subjected to density gradient centrifugation according to ISEV guidelines to obtain apoptotic body-sized extracellular vesicles. Before flow cytometry (FCM) analysis, we used PE-RANK and FITC-Annexin V antibodies to mark osteoclast-derived apoptotic bodies in pelleted vesicles, and added 1 μm and 5 μm polyethylene microspheres to gate 1–5 μm-sized vesicles (Fig. 1D). The FCM results showed that in the process of osteoporosis, the number of apoptotic body-sized extracellular vesicles decreased significantly by more than 50%, as well as osteoclast-derived apoptotic bodies (Fig. 1E). These data declared that the number of osteoclast-derived apoptotic bodies is gradually reduced during osteoporosis and implied that the oscillation of OC-ABs abundance in the bone microenvironment may be associated with the pathological progression of osteoporosis.

### Extraction and identification of osteoclast-derived apoptotic bodies

**Fig. 2:**
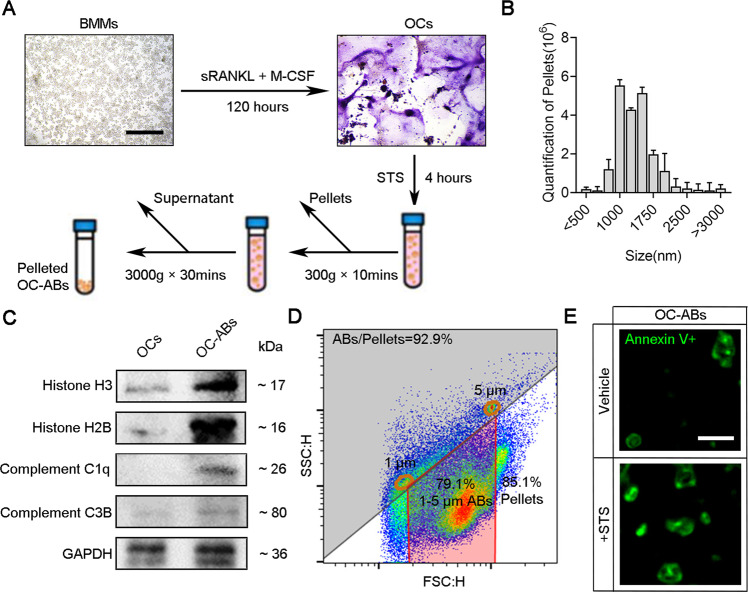
Extraction and identification of osteoclast-derived apoptotic bodies. **A** Schematic diagram of osteoclast apoptosis induction and apoptotic body extraction. **B** Nanoparticle tracking analysis of OC-ABs, *n* = 3. **C** Western blot result of H3, C1QC, H2B, C3B in OCs lysate and pelleted OC-ABs. **D** cytometry analyze result of OC-ABs purity. 1μm and 5 μm diameter beads were used to gate 1–5 μm sized microvesicles. **E** Representative confocal images of OC-ABs (stained with FITC-Annexin V). Scale bar = 2 μm. The data in each panel represent the means ± SD.

To obtain large numbers of pure OC-ABs for investigating its biological function, bone marrow macrophages (BMMs) were incubated by 25 ng/mL M-CSF and 50 ng/mL sRANKL for osteoclastogenesis. Subsequently, staurosporine (STS) treatment was performed to induce the mature osteoclasts apoptosis, and the supernatant was gradient centrifuged to pellet microvesicles considered as OC-ABs (Fig. 2A). For OC-ABs identification, the pelleted OC-ABs were detected by nanoparticle tracking analysis and the particle size was mainly concentrated in the range of 1000–1500 nm (Fig. 2B). The western blot result indicated the enrichment of classical apoptosis body markers in OC-ABs’ total protein including Histone 3 (H3), Histone 2B (H2B), Complement C1q C Chain (C1QC) and Complement 3b(C3B), compared with OCs’ (Fig. 2C). Alternatively, the FCM results showed that the purity of OC-ABs more than 92% within the threshold of 1–5 μm gate screening (Fig. 2D). Under confocal microscope, the apoptotic bodies produced by normal cultured or STS-treated osteoclasts showed consistent vesicle-like structures with diameters ranging from 1–5 μm (Fig. 2E). Taking together, we obtained the pure OC-ABs which could be used in subsequent experiments via STS-treatment in vitro.

### Osteoclast-derived apoptotic bodies have pro-osteogenic effects in vitro

**Fig. 3:**
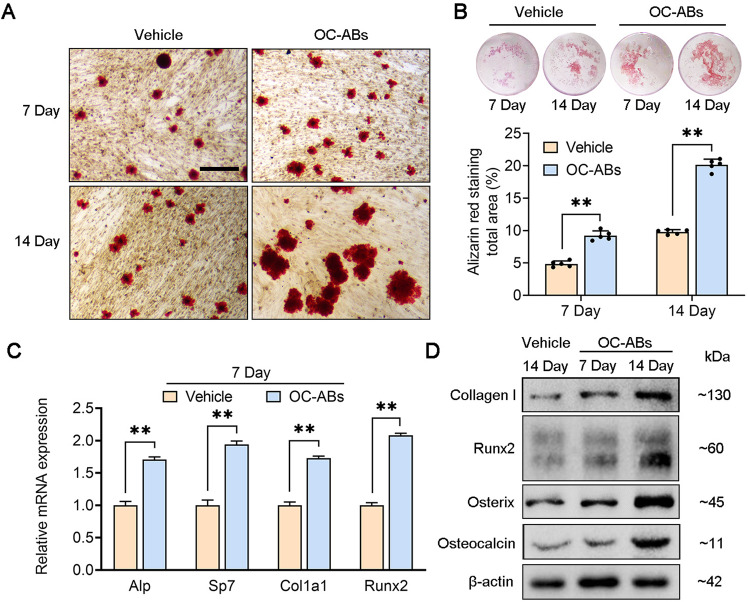
Osteoclast-derived apoptotic bodies have pro-osteogenic effects in vitro. **A** Representative Alizarin Red stained images for each indicated group, scare bar = 100 μm. **B** Representative trans-well images and quantification of mineralized areas, *n* = 5. **C** Real-time PCR results showed the mRNA relative expression of *Alp, Sp7, Col1a1* and *Runx2* between the indicated groups, n = 3. **D** Western blot showed the Collagen I, Runx2, Osterix and Osteocalcin expression of indicated groups. The data in each panel represent the means ± SD. *p* values were obtained by student’s two-tailed unpaired *t* test, *p < 0.05, **p < 0.01, ns = non-significant.

Our previous studies have found that OC-ABs mediate the osteogenic differentiation of bone marrow mesenchymal stem cells in the physiological process of bone remodeling, and maintain the balance of bone formation and bone resorption [24]. Alizarin red staining results showed that the induction medium supplemented with OC-ABs had stronger osteogenic ability in the process of promoting the osteogenic differentiation of BMSCs, which was manifested in the expansion of the mineralized area (Fig. 3A). The results of trans-well images and statistics also showed that the supplementation of OC-ABs could exponentially improve the osteogenic differentiation and mineralization levels of BMSCs (Fig. 3B). At the molecular level, OC-ABs significantly promoted the transcription of osteogenic-related genes (*Alp, Sp7, Col1a1 and Runx2*) and the expression of osteogenic markers (Collagen I, RUNX 2, Osterix and Osteocalcin) in BMSCs (Fig. 3C, D). These findings all emphasized the pro-osteogenic biological function of OC-ABs in vitro and implied the deficiency of OC-ABs would be associated with bone loss in the progression of osteoporosis.

### Membranous RANK of OC-ABs activates the reverse RANKL signaling pathway in bone mesenchymal stem cells

**Fig. 4:**
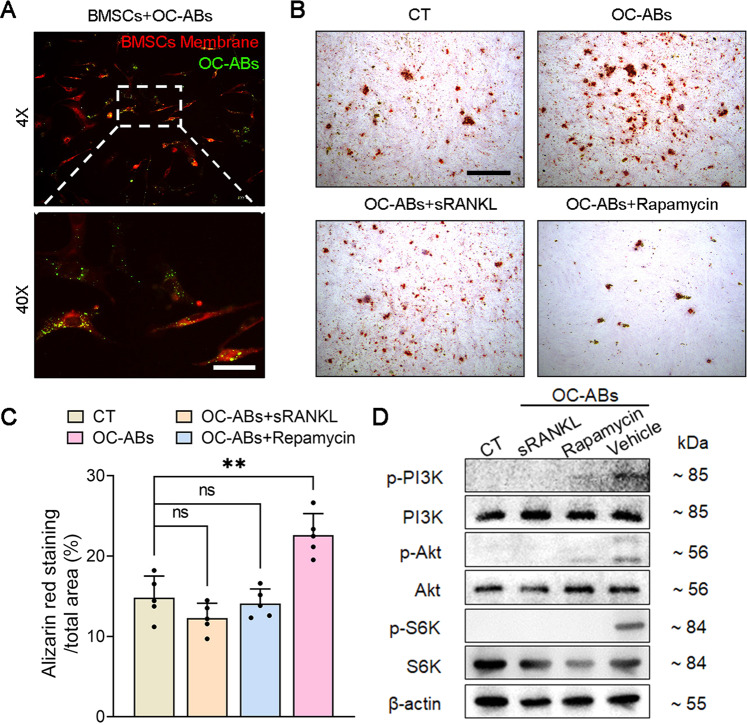
Membrane RANK of OC-ABs activates the reverse signaling pathway in bone mesenchymal stem cells. **A** Fluorescence images showed that OC-ABs were enriched on the cell membrane of BMSCs, scare bar = 30 μm. **B** Representative Alizarin Red stained images for each indicated group, scare bar = 100 μm. **C** Quantification of mineralized areas from indicated group, *n* = 5. **D** Western blot showed the expression levels of mTOR downstream signaling pathway proteins in each indicated group. One-way ANOVA analysis was used to compare multiple sets of data in (**C**), *p < 0.05, **p < 0.01, ns non-significant.

Recently, our group reported that OC-ABs promoted osteogenesis of BMSCs by activating the downstream mTOR pathway via RANKL-mediated reverse signaling [25]. Fluorescence microscopy images showed that OC-ABs were extensively enriched on the surface of BMSCs in the induction medium supplemented with OC-ABs (Fig. 4A). Replacing the supplemented OC-ABs in the induction medium with OC-ABs pre-incubated with sRANKL, the osteogenic differentiation and mineralization of BMSCs did not show similar results as before (Fig. 4B). Furthermore, after adding rapamycin to OC-ABs conditioned medium for blocking mTOR signaling, the mineralized crystals were hardly observed in experimental group (Fig. 4B). Statistical differences in mineralized area also indicated that interference of RANKL reverse signaling or blockade of mTOR signaling pathway directly removed the pro-osteogenic ability of OC-ABs (Fig. 4C). Detecting the downstream marker proteins of mTOR pathway, western blot results also confirmed that OC-ABs mainly achieved their pro-osteogenic biological functions by acting RANK-mediated reverse signaling (Fig. 4D).

### Mice deficient of OC-AB showed decreased bone volume fraction in vivo

**Fig. 5:**
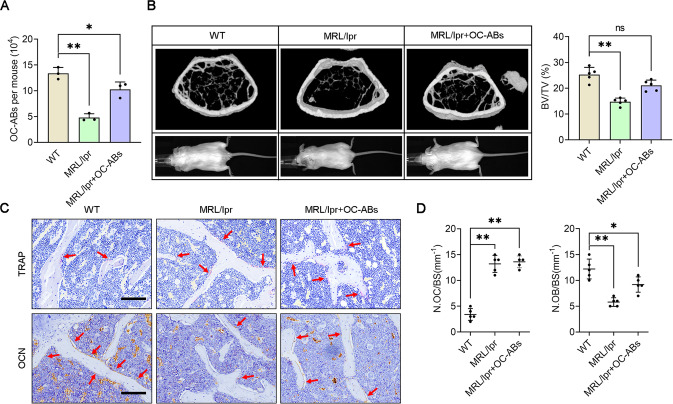
Loss of OC-AB numbers directly alters bone volume fraction in vivo. **A** Quantification of OC-ABs from femoral bone marrow in each indicated mice, *n* = 3. **B** Representative micro-CT images and body size pictures of mice in each group. Quantification of bone volume fraction from indicated mice femoral. **C** Representative TRAP staining images and OCN immunohistochemical images of tibia from the indicated group of mice, scare bar = 100 μm. **D** Quantification of osteoblasts (N.OB/BS) and osteoclasts (N.OC/BS) on bone surface, *n* = 5. One-way ANOVA analysis was used to compare multiple sets of data, *p < 0.05, **p < 0.01, ns non-significant.

To further study the biological function of OC-ABs and highlight the impact of their absence on bone health, Fas-mutated MRL/lpr mouse was used as apoptotic body-deficient model animal for next investigation [27, 28]. We weekly infused OC-ABs into 4-week-old female MRL/lpr mice to restore the abundance of OC-ABs in the bone microenvironment, and 8 weeks later, synchronously extracted femurs from female wild-type mice (WT), OC-ABs reconstructed MRL/lpr mice (MRL/lpr + OC-ABs) and littermates (MRL/lpr) for OC-ABs counting. The FCM results indicated that OC-ABs were deficient in the bone microenvironment of MRL/lpr mice, and weekly tail vein infusion could effectively replenish the abundance of OC-ABs in the bone marrow (Fig. 5A). Subsequently, examining and analyzing the bone mass of the femurs from three groups of mice, we find it interesting that the bone volume fraction of MRL/lpr mice was only about half that of WT mice, while the bone volume fraction of MRL/lpr + OC-ABs mice was roughly the same as WT mice’s (Fig. 5B). We further stained the bone tissue from the three groups of mice, and TRAP staining result showed higher activity of osteoclasts in the bone marrow cavity of MRL/lpr mice and MRL/lpr + OC-ABs mice, likely due to reduced apoptosis (Fig. 5C). On the other hand, osteocalcin (OCN) staining result indicated that the number and thickness of trabecular bone and osteoblast abundance in MRL/lpr mice were significantly reduced, compared with WT mice (Fig. 5C). Notably, reinfusing OC-ABs improved the bone quality of MRL/lpr mice and effectively increased the number of osteoblasts on trabecular surface (Fig. 5C). Statistical analysis showed that the *Fas*-deficiency in MRL/lpr mice resulted in three-fold increase of TRAP^+^ osteoclasts in MRL/lpr mice, and high-activation bone resorption accompanied by inefficient osteogenic capacity worsened the progression of bone loss (Fig. 5D). Meanwhile, the new discovery was that the OC-ABs supplementation restored the abundance of osteoblasts in MRL/lpr mice, which is thought to be beneficial for bone formation against activated bone resorption (Fig. 5D). These findings suggest that although osteoclast-mediated bone resorption directly leads to the reduction of bone mass, the lack of OC-ABs caused by apoptosis inhibition downregulates the osteogenic capacity in bone remodeling and exacerbates the rate of bone loss.

### Systemic infusion of OC-ABs delays osteoporosis progression in OVX mice

**Fig. 6:**
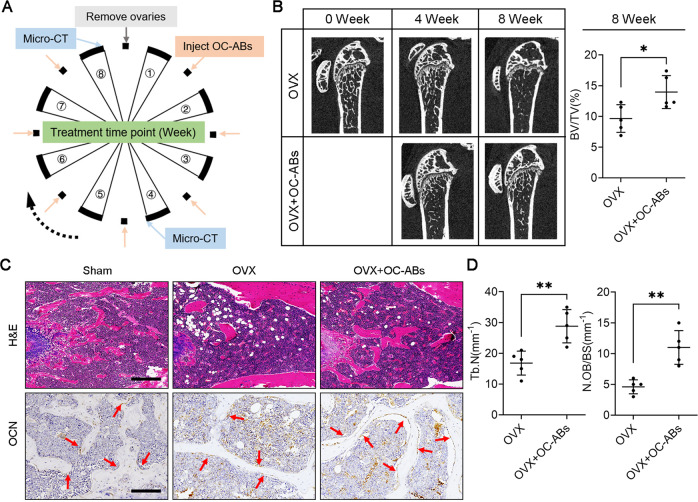
Systemic infusion of OC-ABs delays osteoporosis progression in postmenopausal mice. **A** Experimental design to explore osteoporosis progression under apoptotic body supplementation (infusion via the tail vein, 4 × 10^6^ OC-ABs/each time). **B** Representative micro-CT images and quantification of bone volume fraction from indicated mice femoral. **C** Representative H&E staining images and OCN immunohistochemical images of tibia from the indicated group of mice, scare bar = 100 μm. **D** Quantification of trabecular bone (Tb.N) and osteoblasts (N.OB/BS) on bone surface, *n* = 5. The data in each panel represent the means ± SD. *p* values were obtained by student’s two-tailed unpaired *t* test, *p < 0.05, **p < 0.01, ns non-significant.

To emphasize the importance of OC-ABs in the pathological progression of osteoporosis, we designed rescue experiments in OVX mice and recorded bone-related indicators. In brief, after OVX surgery, we performed weekly OC-ABs infusion in mice, and femur samples were taken for micro-CT scanning and section staining at 4th and 8th weeks (Fig. 6A). The micro-CT data showed that OC-ABs infusion can delay the progression of osteoporosis and save 1/3 of the lost bone volume fraction, compared with OVX mice in the same period (Fig. 6B). Not only that, the results of H&E and OCN staining of bone tissue sections showed that the number and thickness of trabecular bone in mice in the OC-ABs infusion group were significantly increased, as well as the abundance of osteoblasts on the trabecular bone surface (Fig. 6C). Statistics also confirmed that continuous 8-week OC-ABs infusion had a positive effect on delaying bone loss during OVX progression (Fig. 6D). In total, these data suggest that OC-ABs do not exhibit a biological function consistent with the parental osteoclasts enhancing bone resorption in osteoporosis progression, but rather act more as a bone protective factor, striving to correct imbalances in bone remodeling via promoting bone formation.

## Discussion

In this study, we performed OVX in mice establishing a stable osteoporosis model and quantified the OC-ABs from femoral bone marrow using FCM. Compared with wild-type mice, we found that the OC-ABs abundance in the femoral bone marrow from osteoporotic mice was significantly reduced, and the reduction ratio was positively correlated with the degree of bone loss. We further rescued OC-ABs level in apoptotic bodies-deficient MRL/lpr mice and discovered that the scarcity of OC-ABs in vivo was associated with decreased bone mass by evaluating bone volume fraction parameters. Moreover, we revealed that OC-ABs have biological properties of delaying the pathological process of osteoporosis through the experiment that systemic infusing OC-ABs to OVX mice.

In current osteoporosis treatment guidelines, bisphosphonates (BPs) are considered to be the widest selection among therapeutic agents due to their excellent anti-osteoclast resorption effect [29]. Several studies have shown that BPs deposited in the bone are released when osteoclasts destroy the bone unit, and promote the apoptosis of osteoclasts, thereby delaying the loss of bone mass in patients [30]. As a supplement, our study suggests that bisphosphonate-induced OC-ABs may also have pro-osteogenic properties, enhancing the osteogenic capacity of osteoblasts while resisting osteoclast bone resorption, which needs to be confirmed by further experiments with bisphosphonate-induced OC-ABs. In addition, several clinical trials have shown that the rate of bone loss after discontinuation of denosumab (RANKL monoclonal antibody) is significantly higher than that of zoledronic acid and alendronate (one of BPs) [31]. This phenomenon can be explained by our research that even if all drugs act on osteoclasts to achieve anti-bone resorption therapy, the apoptotic bodies generated after bisphosphonate-induced osteoclast apoptosis can still reside in the bone microenvironment against osteopenia for a period of time, while denosumab inhibiting osteoclastogenesis cannot create such a bone protective factor for delaying bone loss.

In recent years, extracellular vesicle-mediated intercellular communication has provided new insights into most biological events, attracting increasing attention in various fields, including but not limited to bone remodeling [32–34]. As far as the research on the crosstalk between extracellular vesicles and osteoporosis is concerned, there have been more than 100 literature reports in the past five years. Most of these studies have found that exosomes derived from different cells can act on bone marrow mesenchymal stem cells and osteoclasts to regulate the balance of bone formation and bone resorption by carrying proteins and non-coding RNAs, thereby affecting the progression of osteoporosis. Studies have shown that apoptosis plays a critical role in pathogenesis and treatment of bone-related diseases. Gap junctions and hemichannels are main communication routes in bone tissue and play a role in both cell survival and death. Bone-related diseases, such as osteonecrosis, can result from apoptosis, which can be prevented by anti-apoptotic strategies such as inhibiting caspases, PARP, and Bcl-2 and inducing the PKB/Akt pathway and IAP family of proteins [35]. Nevertheless, ABs, which also carry abundant biomolecules and are involved in intercellular communication, have received little attention [22, 36]. A very recent review summarized the roles of circulating ABs affecting MSCs and improve the osteoporotic phenotype through various cellular factors, such as AB-derived ubiquitin ligase RNF146 and miR-328-3p [37]. Particularly, RANKL reverse signaling can compensate for the loss of OB-OC coupling signals to prevent decreased bone formation [37]. Although OC-ABs have good biological compatibility, they are difficult to develop into drugs due to their complex contents. The systemic reinfusion of allogeneic OC-ABs in our experiments may have produced abnormal immune responses, masking partly biological functions of OC-ABs. We hope that future engineering solutions for ABs will improve the limitations of the use of OC-ABs in experimental design and achieve the good wishes of preventing, diagnosing and treating osteoporosis [32].

Although Liu et al. found that BMSCs-derived apoptotic bodies can regulate RNF146 in distant MSCs, thereby restoring damaged MSCs function and improving osteopenia in apoptosis-deficient mice, these findings are far from enough to elucidate the biological function of apoptotic bodies in bone remodeling [28]. After all, when using STS to induce apoptotic body production of BMSCs in vivo, it is difficult to avoid the impact on other cells in the bone microenvironment, especially osteoclasts. In addition, for the evaluation of bone mass in MRL/lpr mice, similar to the above research group, we focused on the ABs-deficient in bone marrow, but ignored the immune abnormalities caused by the deletion of the *Fas* in vivo, which may be associated with the low bone mass exhibited by MRL/lpr mice. Herein, our study found that a large number of OC-ABs have a pro-osteogenic effect and can act as a bone protective factor to reduce the rate of bone loss during the pathological progression of osteoporosis, which complement the study of apoptotic bodies in osteopenia. Mechanistically, we were inspired by RANKL reverse signaling and confirmed in multiple previous studies that osteoclast-derived apoptotic bodies can enhance the osteogenic ability of BMSCs by upregulating the expression of mTOR pathway, which same applies to this study [25, 38]. Whereas, it is not comprehensive for revealing the complex intercellular communication between osteoclasts and osteoblasts mediated by apoptotic bodies, needing further research and exploration.

Our data demonstrate a decrease in OC-ABs abundance in the pathological progression of osteoporosis, which seems to contradict the higher numbers of activated TRAP^+^ osteoclasts in bone tissue during the same period. With the deepening of the research, we were inspired by the study of Michelle M. McDonald et al., that the end point of osteoclasts after performing the bone resorption is not necessarily apoptosis [39]. There is a possibility that osteoclasts in the process of osteoporosis can be converted into osteomorphs for dormancy under the fluctuation of RANKL, and studies have also reported that the life cycle of osteoclasts in osteoporosis is longer than that in physiological state. Therefore, the changes in the abundance of osteoclast apoptotic bodies are not completely consistent with the fluctuations in the number of osteoclasts, although further studies are needed to confirm this.

Overall, our findings complement studies of intercellular communication in bone microenvironment, highlight the protective role of OC-ABs in osteoporosis, and attempt to explain the different withdrawal reactions between bisphosphonates and denosumab, providing new ideas for the prevention, diagnosis and treatment of osteoporosis.

## Materials and methods

### Reagents

Antibodies against Histone 3 (bs-17422R), C1QC (bs-11336R), H2B (bs-52099R), C3B (bs-4871R), GAPDH (bs-2188R), β-tubulin (bs-0715R) were all purchased from Bioss Antibodies (Beijing, China). Antibody against RANK (#119805) was obtained from BioLegend (California, USA), while antibody against Annexin-V(#AMM01981G) was obtained from BD Biosciences (New Jersey, USA). Fetal bovine serum (FBS) was purchased from CELLCOOK (Guangzhou, China) and penicillin-streptomycin were purchased from Gibco (Thermo Scientific, MA, USA). Recombinant mouse RANKL was purchased from R&D Systems (Minneapolis, MN, USA). TRAP stain kit was obtained from Sigma-Aldrich (NY, USA). Annexin V and PI Apoptosis Kit (F6012) was purchased from US Everbright® Inc. (Suzhou, China). Staurosporine was purchased from Med Chem Express (New Jersey, USA).

### BMMs extraction and osteoclastogenesis

We extracted the hind limbs of eight-week-old female BALB/c mice and collected bone marrow cell suspensions by repeated marrow cavity flushing. Subsequently, the bone marrow cells were cultured in DMEM medium supplemented with M-CSF for 24 h, and the non-adherent bone marrow mononuclear cells (BMMs) were obtained. For osteoclastogenesis, BMMs were cultured in induction medium (DMEM, 10% FBS, 50 ng/mL RANKL, 25 ng/mL M-CSF) at 37 °C with 5% CO_2_ for 120 h to generate osteoclasts.

### Animal experiment

8-week-old female mice underwent ovary removal surgery. In brief, mice were anesthetized with 4% bromoethane-oxygen gas mixture and maintained at 2% concentration. A 1 cm incision was made in the middle of the abdomen of the mouse to expose the lower poles of the kidneys on both sides. The ovarian glands were determined according to the position of the end of the fallopian tube, and they were removed and ligated to stop the bleeding. Then, 100 μL of 1% penicillin-streptomycin solution was dripped into the abdominal cavity, and the peritoneal layer and the skin layer were sutured. BALB/c used in the experiment were obtained from the Laboratory Animal Center of Third Military Medical University (Chongqing, China) and MRL/lpr mice were purchased from the Jackson Laboratory (Maine, USA). In the experiment to observe the progress of osteoporosis, 45 BALB/c mice were randomly divided into sham operation group (*N* = 27) and Operation Group (*N* = 18). In an experiment to investigate the effect of OC-ABs deficiency on bone mass in mice, 8 BALB/c mice served as wild-type controls, and 16 female MRL/lpr mice were randomly divided into MRL/lpr Group and MRL/lpr + OC-ABs Group. In an experiment to investigate the effect of OC-ABs on delaying the progression of osteoporosis, 25 BALB/c mice were randomly divided into OVX group (*N* = 15) and OVX + OC-ABs Group (*N* = 10), 5 BALB/c mice in OVX group were killed at 1 day after operation and taken as blank control. In the animal experiment, each mouse was numbered to achieve single-blind. Numbers, instead of group names, are marked on test tubes or slices containing samples to ensure the examiner blinded. All experimental protocols were reviewed and approved by the Institutional Animal Care and Use Committee of Third Military Medical University, and the mice were euthanized according to the AVMA Guidelines for the Euthanasia of Animals.

### Apoptotic body extraction and identification

After the formation of osteoclasts, the cell supernatant was removed, 5 μM STS in DMEM medium was added, and the cells were further cultured at 37 °C for 4 h to induce osteoclast apoptosis. The culture medium produced after osteoclast apoptosis was collected, centrifuged at 300 × *g* for 5 min to precipitate cells and debris and removed, and the supernatant was centrifuged at 3000 × *g* for 30 min to precipitate apoptotic bodies. Apoptotic bodies were resuspended and washed in PBS, and re-pelleted by centrifugation at 3000 × *g* for 30 min. All centrifugation operations were under 4 °C and performed by using Optima XE-90 (Beckman Coulter). For identification, apoptotic body suspensions were incubated with FITC-Annexin V antibody for 15 min in the dark, after which they were dropped into confocal dishes and visualized using confocal microscopy (Zeiss LSM-800, Germany). The purity of apoptotic body suspensions supplemented with standard size polyethylene microspheres (#M122073& #M122077, Aladdin, Shanghai, China) was examined by flow cytometry (CytoFLEX, Beckman, USA). Western blot was used to detect apoptotic body markers C1QC, C3B, H3 and H2B.

### H&E and TRAP staining

The extracted hindlimbs of mice were fixed in 4% paraformaldehyde for 72 h and then soaked in EDTA decalcification solution for 4 weeks. The decalcified tissue was graded dehydrated and then embedded in paraffin. Subsequently, 5 μm thick sections were made for staining by using Leica microtome (RM2235, Leica Biosystems, Germany). The sections were immersed in hematoxylin staining solution for 1 min, rinsed with water for 30 s, and differentiated with 1% hydrochloric acid alcohol for 10 s. Sections were immersed in eosin staining solution again for 3 min and rinsed with water for 30 s. The slides were sealed with neutral resin after gradient dehydration and observed under microscope. For TRAP staining, bone tissue sections were stained with TRAP staining kit (CS0740, Sigma, Shanghai, China), and the method steps were referred to the reagent manufacturer’s instructions. TRAP staining of osteoclast was also used the above-mentioned kit. The images were acquired and analyzed by the ZEISS Axio Scan-Z1 Fully Automatic Digital Slide Scanning System (Carl Zeiss, Germany).

### Osteocalcin staining

Sections were gradient dewaxed and blocked in 3% H_2_O_2_ for 15 min, then the sections were immersed in sodium citrate antigen retrieval solution at 95 °C for 10 min. After 15 min of incubation in immunohistochemical blocking solution, sections were incubated with diluted rabbit anti-OCN (1:200, diluted in blocking solution) for 2 h at room temperature. Further, the sections were washed 3 times with PBS and incubated with horseradish peroxidase-conjugated secondary antibody for 2 h, and then DAB chromogenic reagent was added dropwise for observation under a microscope. The images were acquired by the ZEISS Axio Scan-Z1 Fully Automatic Digital Slide Scanning System and analyzed by ImageJ v.1.8.0 software.

### Osteogenic induction and alizarin red staining

We extracted the hind limbs of eight-week-old female BALB/c mice and collected bone marrow cell suspensions by repeated marrow cavity flushing. Subsequently, the bone marrow cells were cultured in a-MEM medium containing 10% FBS for 8 h to obtained adherent bone marrow mesenchymal stem cells (BMSCs). 1 × 10^5^ BMSCs were seeded into 24-well plates and cultured with basal medium (a-MEM supplemented with 10% FBS) for proliferation. When the cell confluence reaches more than 80%, basal medium was changed to osteogenic induction medium (100 mL a-MEM medium supplemented with 10% FBS, 10 μL 1 mM Dexamethasone, 500 μL 10 mM Vitamin C and 1 mL 1 M β-Glycerophosphate) for continue culture in an incubator at 37 °C with 5% CO_2_. For the observation of calcium salt deposition, cells were fixed with 4% paraformaldehyde for 20 min and washed 3 times with PBS. Cells were covered with alizarin red staining solution (ALIR-10001, OriCell®, Suzhou, China) and protected from light, incubated at room temperature for 30 min and washed with PBS three times. The images were acquired by the Leica DMIL microscopic imaging system (Carl Zeiss, Germany) and the mineralized area was analyzed by ImageJ v.1.8.0 software.

### Micro-CT

Images of the femur were acquired using a Bruker MicroCT Skyscan 1272 system (Kontich, Belgium) with a resolution of 8.0 μm voxel size and data reconstruction was performed using Nrecon (Kontich, Belgium). Select regions of interest (ROI) and acquire 2D images in DataViewer 1.5.6.2 (Bruker, Kontich, Belgium). The bone volume fraction and other data of the ROI were obtained by CTAn 1.20.8.0 (Bruker, Kontich, Belgium) software.

### Real-time polymerase chain reaction (PCR)

Total RNA was isolated from the cultures using the RNA-Quick Purification Kit (YiShanBiotech, Shanghai, China) according to the manufacturer’s instructions. The cDNA was synthesized using SuperScript III (Life Technologies). The real-time PCR was performed using SYBR Green Supermix (Toyobo) and gene-specific primers. The primers included *Alp* forward 5′-AACCCAGACACAAGCATTCC-3′; reverse 5′-GAGACATTTTCCCGTTCACC-3′. *Sp7* forward 5′- ATGGCGTCCTCTCTGCTTG-3′; reverse 5′- TGAAAGGTCAGCGTATGGCTT-3′. *Col1a1* forward 5′-GCTCCTCTTAGGGGCCACT-3′; reverse 5′-ATTGGGGACCCTTAGGCCAT-3′. *Runx2* forward 5′-ATGCTTCATTCGCCTCACAAA-3′; reverse 5′-GCACTCACTGACTCGGTTGG-3′. *GAPDH* forward 5′-TGGATTTGGACGCATTGGTC-3′; 5′-TTTGCACTGGTACGTGTTGAT-3′. *GAPDH* was used as an endogenous control for osteoclasts. Real-time PCR was detected on a CFX96™ Real-Time PCR System (Bio-Rad).

### Western blot analysis

Cells or ABs were lysed in RIPA lysis buffer (CWBIO, Beijing, China) mixing with protease phosphatase inhibitor (Beyotime Biotechnology, Shanghai, China). The lysis system was centrifuged at 12,000 × *g* for 15 min to obtain the supernatant after incubated on ice for 30 min. The BCA protein assay kit (Beyotime Biotechnology, Jiangsu, China) was used to detect the concentration of proteins. 50 μg protein samples were diluted in loading buffer (Beyotime Biotechnology, Jiangsu, China) and electrophoresed, followed by transferred onto polyvinylidene fluoride membranes (ImmobilonTM-PSQ Membranes, Sigma-Aldrich, China) and blocked in 5% bovine serum albumin (BSA). Protein on membranes were incubated with anti AKT, anti p-AKT, anti PI3K, anti p-PI3K, anti S6K, anti p-S6K and anti β-actin (1:1000, diluted in 5% BSA) antibodies for 12 h at 4 °C. After washing in Tris Buffered Saline Tween buffer three times, secondary antibodies linked HRP (1:10,000, diluted in 5% BSA) against primary antibodies were used to incubate membranes. Super ECL Plus (Biosharp, Anhui, China) and the ChemiDoc XRS + gel imaging system (BioRad) were used to detect chemiluminescent signals.

### Statistics

All experiments were repeated at least three times carried out with at least three biological replicates. Comparisons between two groups were analyzed by using independent unpaired two-tailed Student’s *t*-tests. One-way ANOVA analysis was used to compare multiple sets of data. GraphPad Prism 8.0 software was used for statistical analysis. *P* values < 0.05 and *P* values < 0.01 were used to represent the significance of the difference. The error bars in the figures represent the standard deviation (SD).

## Supplementary information

original blots

## Data Availability

The data are available from the corresponding author (lance.douce@gmail.com) on reasonable request.
