# Supplementary material for: Reduced osteoclast-derived apoptotic bodies in bone marrow characterizes the pathological progression of osteoporosis
Source: Cell Death Discov. 2023 Apr 26;9:135. doi: 10.1038/s41420-023-01434-w (PMC10130088; doi:10.1038/s41420-023-01434-w)

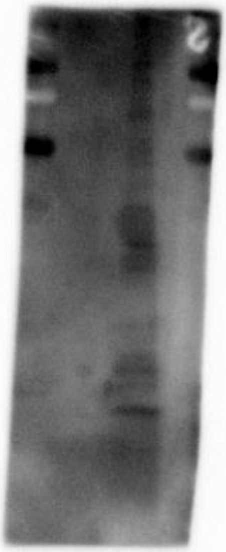

Fig.2 C1q

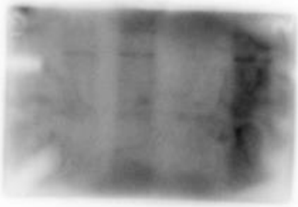

Fig.2 C3B

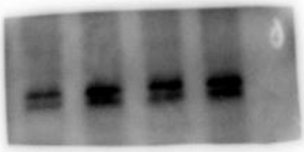

Fig.2 GAPDH

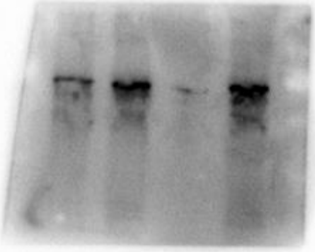

Fig.2 H2B

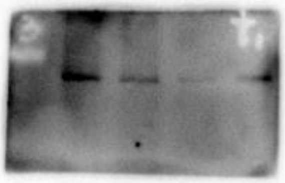

Fig.2 H3

Fig.3 β-actin

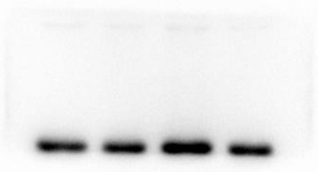

Fig.3 Collagen1

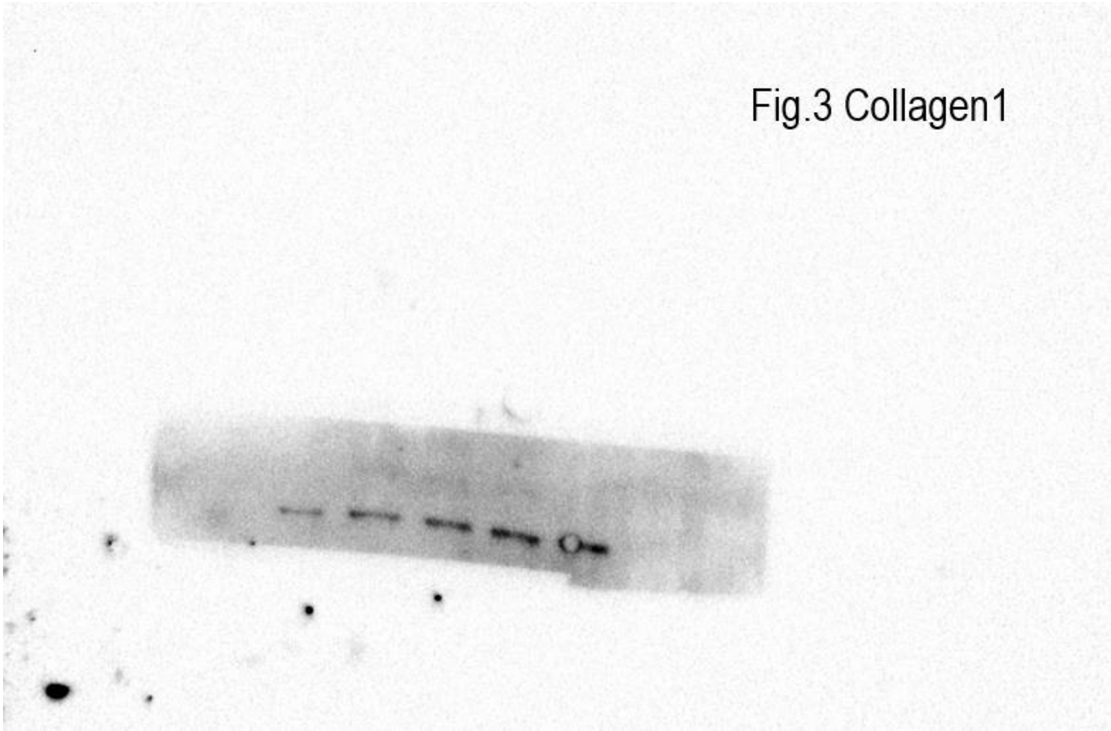

Fig.3 Osteocalcin

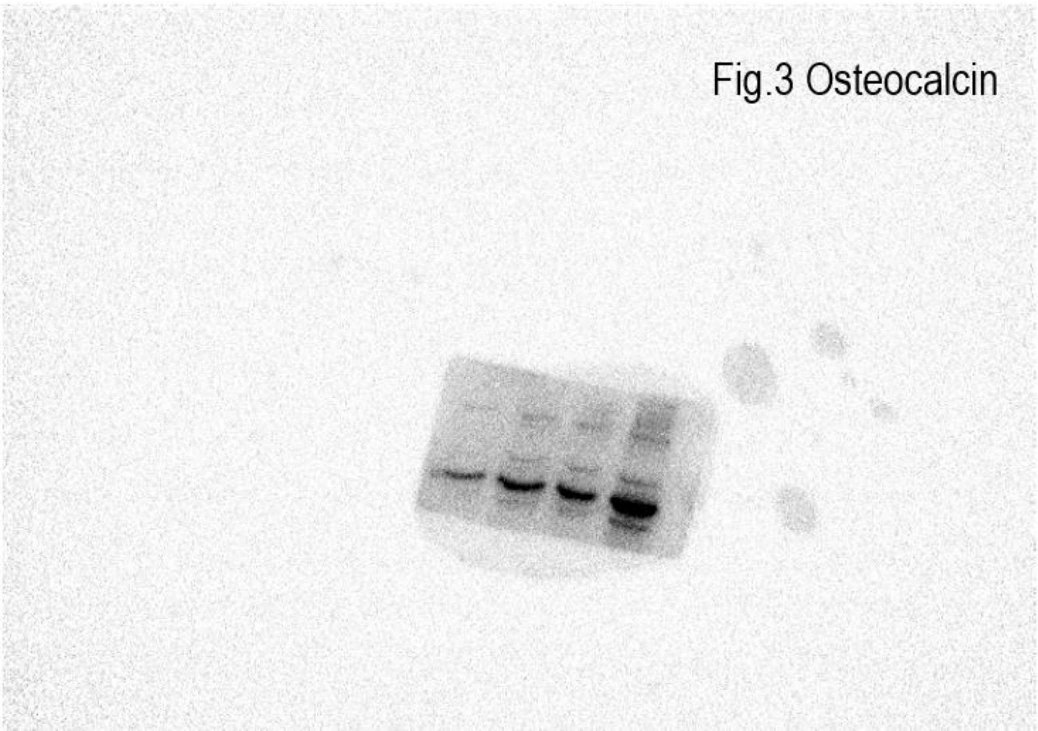

Fig.3 Osterix

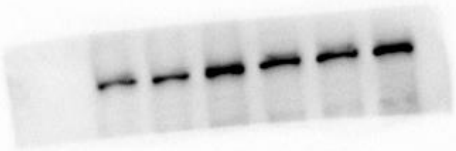

Fig.3 RUNX2

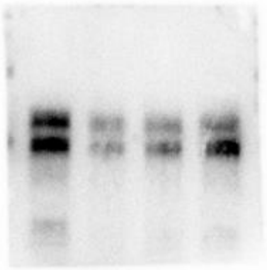

Fig.4 AKT

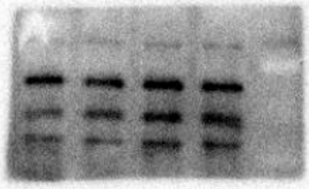

Fig.4 β-actin

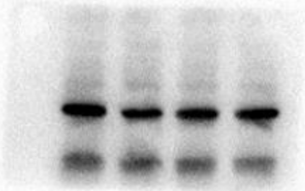

Fig. 4 p-AKT

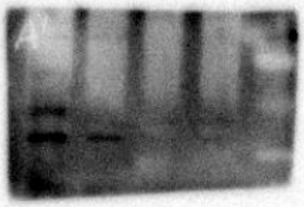

Fig.4 PI3K

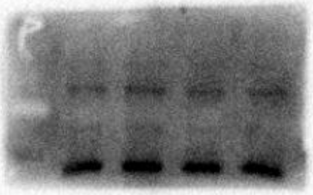

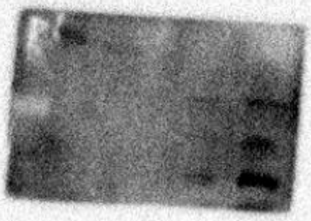

Fig.4 p-PI3K

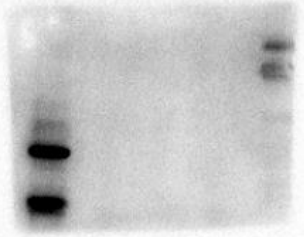

Fig.4 p-S6K

Fig.4 S6K

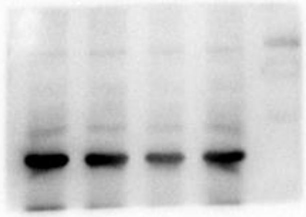

Supplement: Supplementary file 1 — original blots [file 41420_2023_1434_MOESM1_ESM.pdf]
